# Nonlinear expression and visualization of nonmetric relationships in genetic diseases and microbiome data

**DOI:** 10.1186/s12859-018-2537-z

**Published:** 2018-12-21

**Authors:** Xianchao Zhu, Xianjun Shen, Xingpeng Jiang, Kaiping Wei, Tingting He, Yuanyuan Ma, Jiaqi Liu, Xiaohua Hu

**Affiliations:** 1 School of Computer, Central China Normal University, Wuhan, China; 2 College of Computing and Informatics, Drexel University, Philadelphia, PA 19104 USA

**Keywords:** Multiple maps t-SNE, Data visualization, Non-metric similarities, Nesterov momentum

## Abstract

**Background:**

The traditional methods of visualizing high-dimensional data objects in low-dimensional metric spaces are subject to the basic limitations of metric space. These limitations result in multidimensional scaling that fails to faithfully represent non-metric similarity data.

**Results:**

Multiple maps t-SNE (mm-tSNE) has drawn much attention due to the construction of multiple mappings in low-dimensional space to visualize the non-metric pairwise similarity to eliminate the limitations of a single metric map. mm-tSNE regularization combines the intrinsic geometry between data points in a high-dimensional space. The weight of data points on each map is used as the regularization parameter of the manifold, so the weights of similar data points on the same map are also as close as possible. However, these methods use standard momentum methods to calculate parameters of gradient at each iteration, which may lead to erroneous gradient search directions so that the target loss function fails to achieve a better local minimum. In this article, we use a Nesterov momentum method to learn the target loss function and correct each gradient update by looking back at the previous gradient in the candidate search direction.

By using indirect second-order information, the algorithm obtains faster convergence than the original algorithm. To further evaluate our approach from a comparative perspective, we conducted experiments on several datasets including social network data, phenotype similarity data, and microbiomic data.

**Conclusions:**

The experimental results show that the proposed method achieves better results than several versions of mm-tSNE based on three evaluation indicators including the neighborhood preservation ratio (NPR), error rate and time complexity.

## Background

A large number of studies have shown that genetic diseases with overlapping phenotypes are closely related to function-related gene mutations [1, 2]. From another perspective, there are similar pathophysiological mechanisms between different clinical features and genetic diseases [3, 4]. In addition, classical methods of dimensionality reduction and visualization of data have been applied to the analysis of microbial data [5]. However, generally speaking, the integration and analysis of microbiome big data are still in its preliminary stage. There are currently no effective integration techniques and visualization methods to exploit microbiome big data. Some studies have focused on established mathematical models that exploit the complicated correlations between phenotypes and genotypes in isomeric genomic datasets such as genetic expression data, gene ontology annotations [6], and protein-protein interaction networks [7, 8]. In addition, some studies prove that non-metric attributes are important features of microbial data [9]. Researching the associations between diseases not only helps us to discover their mutual hereditary basis [10], but also provides us new insights into the molecular circadian mechanisms [11] and prospective drug target studies [12] Each person’s gut microbiota has a dominant flora in the intestine and can be divided into three different “intestinal types” based on the characteristics of the human intestine. This finding can help us discover the relationship between drugs, diet, microbes and the body in different states of health and disease [13]. These microbes distributed in different parts of the body play a vital role in our health. Lowering the dimensions of data and extracting useful information from data in the analysis of microbiome big data, with the help of statistics and pattern recognition, the structure and characteristics of the microbial community could be analyzed; new biological hypothesis could be proposed and examined.

**Fig. 1:**
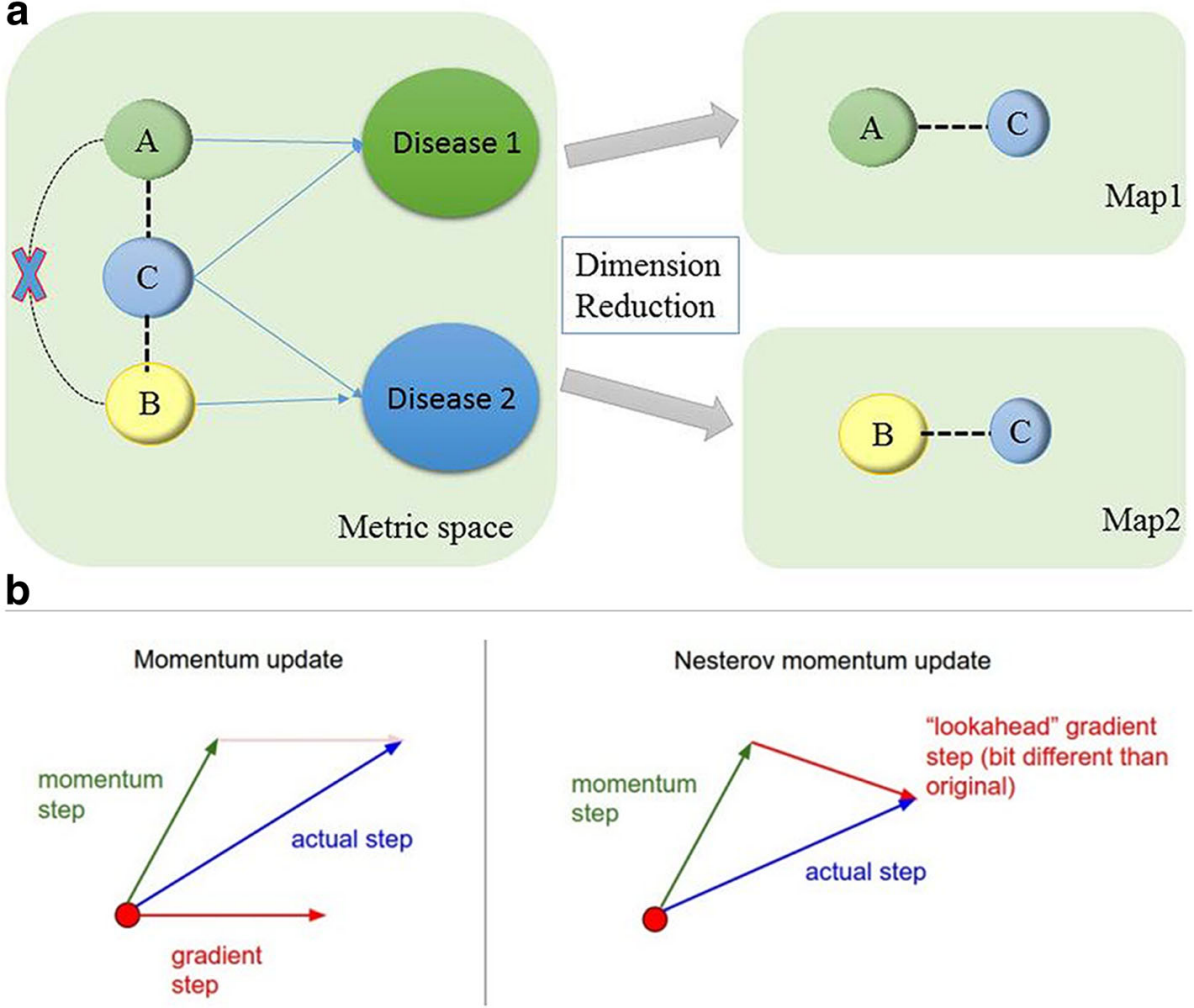
The interpretation of non-metric space similarity and the difference between Nesterov momentum and standard momentum

Before performing computational tasks on a large amount of data, to conduct preliminary visualization and exploration at first will helps us understand this task intuitively. By visualizing the relationships between disease phenotypes, we may gain new insights into the relationships between genes and disease. The conventional method of dimensionality reduction visualizes high-dimensional space objects into two-dimensional or three-dimensional metric space by constructing a single map in low-dimensional space [14]. However, this visualization method suffers from the basic limitations of the metric space. The main limitation of metric space comes from the triangular inequality criterion. For example, from a biological point of view, if phenotype A is associated with phenotype B in the metric space and phenotype B is associated with phenotype C, logically, phenotype A should be associated with phenotype C. As a matter of fact, this restriction is most likely to be ruined by the implicit structure of similarity data. Because these diseases may be interrelated in different categories, they may have overlapping phenotypes in which a cluster of phenotypes may belong to disparate illness categories. The mm-tSNE [15] can properly model non-transitive similarities by assign a significance weight to each point in disparate maps. For example, we imbed three instance phenotypes A, B, and C into two maps in low dimensional space (see Fig.1 (a)), mm-tSNE assigns a significance weight of 1 to the phenotype A on the first map, assign an importance weight 1 for the phenotype B in the second map and assign to the phenotype C a significance weight in both maps is 0.5. As a result, the pairs of similarities between phenotype A and B is 0. The mm-tSNE approach breaks down the nature of metric-space transitivity similarities by visualizing data points into multiple maps [15]. Nevertheless, mm-tSNE may have some drawbacks, that is, the data points with high significance weights in the uniform map do not accord with the uniform cluster structure. That adds to the difficulty of explaining the implication of each and every map. The mm-tSNE regularization [16] improves the mm-tSNE by introducing the Laplacian penalty term in the target loss function. The Laplacian penalty term has been widely applied to many machine learning models [17, 18]. Compared with mm-tSNE, a preponderance of mm-tSNE regularization is that it adopts clustering structure of variate and offers more sparsity for parameter estimation. These methods use standard momentum updates [19] to evaluate point of the gradient at each iteration. But sometimes the gradient of the previous update is wrong, it would make the current update jump high, which leads to excessive oscillation. This article is an extended version of the mm-tSNE regularization based on NAG from an earlier conference publication [20]. In contrast to these previous papers, this article: (1) contains more detailed technical and experimental descriptions; and (2) includes additional experimental results on some microbial datasets. In this article, we use a Nesterov momentum method [21, 22] to learn the target loss function and correct each gradient update by looking back at the previous gradient in the candidate search direction. The key difference between standard momentum and Nesterov momentum is that standard momentum calculates the gradient before the velocity is applied, while Nesterov momentum calculates the gradient after doing so. Therefore, the calibration gradient can be corrected faster and more accurately. This benign-looking difference seems to allow Nesterov momentum to change velocity in a quicker and more responsive way, letting it behave more stable than momentum in many situations, especially for higher values of momentum coefficient. By indirectly using the information of the second order, the Nesterov momentum method achieves a better convergence rate than the momentum method and further reduces the error rate of the loss function. The results of the present study indicate that the proposed method can obtain comparable performance compared with the original methods and provide a better data visualization framework.

## Methods

### T-distributed stochastic neighborhood embedding (t-SNE)

t-Distributed Stochastic Neighborhood Embedding (t-SNE) is a classical multi-dimensional scaling technique [23] It is a non-linear mapping method based on the early work of Stochastic Neighbor Embedding [24]. As data points are mapped from high-dimensional space to low-dimensional space, the distances between data points are maintained and local information and global information are preserved. This method has been applied to the visualization of data in many fields such as literature [25], linguistic data [26], and breast cancer CADx imaging data [27]. In t-SNE, the similarities amongst data points are modeled by probability metrics different from the Euclidean distance decision. The paired distances between data points in a high-dimensional space are transformed by Gaussian distribution into probability distances *p*_*ij*_ to represent the similarities between data points:

1 $$ {\mathrm{p}}_{ij}=\frac{\exp \left(-\left\Vert {x}_i-{x}_j\right\Vert /2{\sigma}^2\right)}{\sum_k{\sum}_{l\ne \mathrm{k}}\exp \left(-\left\Vert {x}_i-{x}_j\right\Vert /2{\sigma}^2\right)}, for\forall \mathrm{i}\forall \mathrm{j}:\mathrm{i}\ne \mathrm{j}. $$

The aim of t-SNE is to calculate and retain the probabilistic of distances between all object points in low-dimensional space. In t-SNE, the two or three-dimensional “metric space” is defined as a long-tailed distribution *Q*_*ij*_ that centers at each and every point, for purposing of avoiding the “crowding problem [23]”. The paired distances between data points in a low dimensional space is transformed into a probability distance *q*_*ij*_ by t-distribution to represent the similarities between data points:

2 $$ {\mathrm{q}}_{ij}=\frac{{\left(1+{\left\Vert {y}_i-{y}_j\right\Vert}^2\right)}^{-1}}{\sum_{\mathrm{k}}{\sum}_{l\ne k}{\left(1+{\left\Vert {y}_i-{y}_j\right\Vert}^2\right)}^{-1}}, for\forall \mathrm{i}\forall \mathrm{j}:\mathrm{i}\ne \mathrm{j}. $$

The difference between the similarity *q*_*ij*_ in the low-dimensional space and the similarity *p*_*ij*_ in the high-dimensional space is measured by calculating the KL divergence between the joint distributions *P* and *Q*:

3 $$ C= KL\left(P\left\Vert Q\right.\right)={\sum}_{\mathrm{i}}{\sum}_{j\ne \mathrm{i}}{p}_{ij}\log \frac{p_{ij}}{q_{ij}}. $$

### Multiple maps t-SNE

mm-tSNE is a variant of the t-SNE method that breaks down the traditional limitations of a single metric map by constructing multiple mappings *M* in a low-dimensional space to visualize pairwise similarities in non-metric spaces.

Multiple maps t-SNE constructs *M* maps in low dimensional space, where each map contains *N* data points. In the map with index m, the data point with index *i* has an importance weight ${\pi}_i^{(m)}$, which represents the importance of data point *i* in map *M*, and the sum of the weights of data point *i* in all maps is equal to 1. Therefore, the pairwise similarity *q*_*ij*_ between data points in a low-dimensional space is measured by a weighted sum of pairwise similarities between data points *i* and *j* in all the maps. Its mathematical definition is as follows:

4 $$ {q}_{ij}=\frac{\sum_m{\pi}_i^m{\pi}_j^m{\left(1+{\left\Vert {y}_i^{(m)}-{y}_j^m\right\Vert}^2\right)}^{-1}}{\sum_{m^{\prime }}{\sum}_{k\ne l}{\pi}_k^{\left({m}^{\prime}\right)}{\pi}_l^{\left({m}^{\prime}\right)}{\left(1+{\left\Vert {y}_k^{\left({m}^{\prime}\right)}-{y}_l^{\left({m}^{\prime}\right)}\right\Vert}^2\right)}^{-1}}\  for\forall i\forall j:i\ne j, $$

where ${y}_i^{(m)}$ indicates that the data point *i* in the high-dimensional space is mapped to the m map in the low-dimensional space. Since it is more difficult to directly calculate the parameter ${\pi}_i^{(m)}$. In order to simplify the calculation, the weight of importance ${\pi}_i^{(m)}$ is obtained by calculating the unconstrained ${\omega}_i^{(m)}$:

5 $$ {\pi}_i^{(m)}=\frac{e^{-{\omega}_i^{(m)}}}{\sum_{m^{\prime }}{e}^{-{\omega}_i^{m^{\prime }}}}. $$

The objective loss function has the uniform form as Eq. 3, but the cost function minimum is calculated by the location of the point ${y}_i^{(m)}$ in all relevant metric maps and the associated unrestrained weight ${\omega}_i^{(m)}$.

### Multiple maps t-SNE with Laplacian regularization

Multiple maps t-SNE with Laplacian regularization (mm-tSNE regularization) alleviates the problem that the higher-weighted data points in the uniform map do not accord with the uniform clustering structure by adding Laplacian penalties to the original mm-tSNE cost function *C (Y)*.

6 $$ C(Y)= KL\left(P\left\Vert Q\right.\right)=\left(1-\lambda \right){\sum}_i{\sum}_{j\ne i}{p}_{ij}\log \frac{p_{ij}}{q_{ij}}+{\lambda \pi}^T L\pi, $$

where *L* = (*diag*(∑_*j*_*p*_*ij*_) − *P*_*ij*_).

The gradient about the mapping point ${y}_i^{(m)}$ in the low-dimensional space is calculated by the following equation:

7 $$ \frac{\partial C(Y)}{\partial {y}_i^{(m)}}=4\left(1-\lambda \right){\sum}_j\frac{\partial C(Y)}{\partial {d}_{ij}^{(m)}}\left({y}_i^{(m)}-{y}_j^{(m)}\right), $$

where $$ {\mathrm{d}}_{ij}^{(m)}={\left\Vert {y}_i^{(m)}-{y}_j^{(m)}\right\Vert}^2 $$.

The gradient about the weights ${\omega}_i^{(m)}$ in the low-dimensional space is calculated by the following equation:

8 $$ \frac{\partial C(Y)}{\partial {\pi}_i^{(m)}}={\sum}_j\left(\frac{2}{q_{ij}Z}\left({p}_{ij}-{q}_{ij}\right)\right){\pi}_j^{(m)}{\left(1+{d}_{ij}^{(m)}\right)}^{-1}+\lambda L\pi, $$

where $$ Z={\sum}_k{\sum}_{l\ne k}{\sum}_{m^{\prime }}{\pi}_i^{m^{\prime }}{\pi}_k^{m^{\prime }}\left(1+{d}_{kl}^{m^{\prime }}\right) $$.

Mathematically, the gradient update of the momentum item is given by the following equation:

9 $$ {\nu}^{(t)}={\gamma \nu}^{\left(t-1\right)}-\eta \frac{\partial C(Y)}{\partial Y}, $$

10 $$ Y=Y+{\nu}^{(t)}, $$

where *Y* are the model parameters, the velocity is *v*^(*t*)^, the momentum coefficient is *γ* ∈ [0, 1] and *η* is the learning rate at iteration t, $\frac{\partial C(Y)}{\partial Y}$ is the gradient.

### Simplified Nesterov momentum

Nesterov momentum [21, 22] is a first-order optimization method to improve stability and convergence of regular gradient descent. The algorithm update rules are as follows [28, 29]:

11 $$ {v}^{(t)}={\mu}^{\left(t-1\right)}{v}^{\left(t-1\right)}-{\varepsilon}^{\left(t-1\right)}\nabla f\left({\theta}^{\left(t-1\right)}+{\mu}^{\left(t-1\right)}{v}^{\left(t-1\right)}\right), $$

12 $$ {\theta}^{(t)}={\theta}^{\left(t-1\right)}+{v}^{(t)}, $$

where *θ*_*t*_ are the model parameters, the velocity is *v*^(*t*)^, *μ*^(*t*)^ ∈ [0, 1] is the momentum coefficient and *ε*^(*t*)^ > 0 is the learning rate at iteration t, *f*(*θ*) is the objective function and *∇f*(*θ*^′^) is a shorthand notation for the gradient $\frac{\partial f\left(\theta \right)}{\partial \theta}\left|\theta ={\theta}^{\prime}\right.$.

The equivalent form is as follows:

13 $$ \hat{v^{(t)}}={\mu}^{\left(t-1\right)}\hat{v^{\left(t-1\right)}}\hbox{-} {\varepsilon}^{\left(t-1\right)}\nabla f\left(\hat{\theta^{\left(t-1\right)}}\right)\hbox{-} {\varepsilon}^{\left(t-1\right)}{\mu}^{\left(t-1\right)}\left[\nabla f\left(\hat{\theta^{\left(t-1\right)}}\right)-\nabla f\left(\hat{\theta^{\left(t-2\right)}}\right)\right]. $$

14 $$ \hat{\theta^{(t)}}=\hat{\theta^{\left(t-1\right)}}+\hat{v^{(t)}}. $$

Different from the momentum term, Nesterov momentum renews the parameter vector at some position*θ*^(*t*)^, which depends on *μ*^(*t* − 1)^*ν*^(*t* − 1)^ as well as in the last momentum update of the current parameter position. The gradient correction to the velocity*v*_*t*_, with the Nesterov momentum, is calculated at point *θ*^(*t*)^ + *μ*^(*t* − 1)^*v*^(*t* − 1)^, and if *μ*^(*t* − 1)^*v*^(*t* − 1)^ is an even worse update, *∇f*(*θ*^(*t* − 1)^ + *μ*^(*t* − 1)^*v*^(*t* − 1)^) will point reversely *θ*^(*t*)^ more forcefully than the gradient computed at *θ*^(*t*)^, hence providing a larger and more timely correction to *v*^(*t*)^. Fig. 1 (b) illustrates the geometric significance of this phenomenon. With the equivalent form of Nesterov momentum, we can observe the difference between Nesterov momentum and standard momentum. The direction of this update has increased by an amount of ${\mu}^{\left(t-1\right)}\left[\nabla f\left(\hat{\theta^{\left(t-1\right)}}\right)-\nabla f\left(\hat{\theta^{\left(t-2\right)}}\right)\right]$, the change is essentially an approximation of the second order of the objective function. Since Nesterov momentum uses the second-order information of the objective function, the Nesterov momentum is more efficient than the standard momentum term in modifying the large and undue velocity in each iteration, which makes it run faster than the momentum method, and can further reduce the error rate of the loss function.

### Multiple maps t-SNE regularization based on Nesterov momentum

In this article, unlike the original several versions of mm-tSNE, we use the Nesterov momentum method to optimize the target loss function, which lets the loss function reach the optimal value better and faster and obtain a higher neighborhood preservation ratio.

The learning algorithm is as follows:

15 $$ {\nu}^{(t)}={\gamma \nu}^{\left(t-1\right)}-\eta \frac{\partial C(Y)}{\partial}\left(Y+{\gamma \nu}^{\left(t-1\right)}\right). $$

16 $$ Y=Y+{\nu}^{(t)}, $$

where *Y* represents the model parameter to be optimized, *ν*^(*t*)^ represents the velocity of the *i* iteration, *γ* ∈ [0, 1] represents the momentum coefficient, *η* represents the learning rate for the *i* iteration, and $\frac{\partial C(Y)}{\partial Y}$ represents the gradient.

#### Datasets

To assess the performance of our approach, we apply our method to several datasets, including phenotypic similarity dataset and microbial dataset. The microbial dataset consisted of 6313 orthologous proteins which are from 345 individual intestinal microorganisms [30]. After data preprocessing, a similarity matrix of 1299 KOs is finally obtained. The phenotypic similarities come from the Online Mendelian Inheritance in Man (OMIM) database [31, 32], which contains 1025 phenotypes related to 21 diseases, respectively, according to the disease classification information from the Human Disease Network [8]. At them in the middle, the value of similarity less than 0.5 is filtered out.

#### Evaluation indicators

##### Neighborhood preservation ratio

The ideal state for dimensionality reduction visualization is that the neighboring point of the sample point *x*_*i*_ in the high-dimensional space is exactly the same as its neighboring point in the low-dimensional space*y*_*i*_. That is, it is assumed that the neighboring points around the sample point *x*_*i*_ pass through the high-dimensional space. After the dimensional method is projected into a two-dimensional space, the neighboring points around*y*_*i*_coincide with the high-dimensional space. The neighborhood preservation ratio is a measure proposed by Laurens van der Maaten [15], which measures similarities in the high-dimensional space are preserved in the low-dimensional space by the mm-tSNE method. For each data point *i*, we choose its *k* highest p_*ij*_-values in the high-dimensional space as its *k* nearest neighbors (*N*^*i1*^ for short), and select the k highest q_*ij*_-values in the low-dimensional space as its *k* nearest neighbors (*N*^*i2*^ for short). By calculating the intersection of *N*^*i1*^ and *N*^*i2*^, it can be determined whether the reduced-dimensional visualization method used can maintain the distribution of neighboring points of data in high-dimensional space. Therefore, NPR indicates the average ratio of the number of neighbors to be saved.

17 $$ NPR=\frac{1}{n}{\sum}_{i=1}^n\frac{\left|{N}^{i1}\cap {N}^{i2}\right|}{k}, $$

where |*N*^i1^ ∩ *N*^*i*2^| is the number of points that common points in high-dimensional space and low-dimensional space and *n* represent the total number of visualized target data points.

##### Error rate

The error rate represents the cost of using the *KL* divergence method to model the difference between the *Q* distribution and the *P* distribution.

##### Time complexity

The time complexity of the algorithm is measured by the number of times the basic operations are repeated.

## Results

**Fig. 2:**
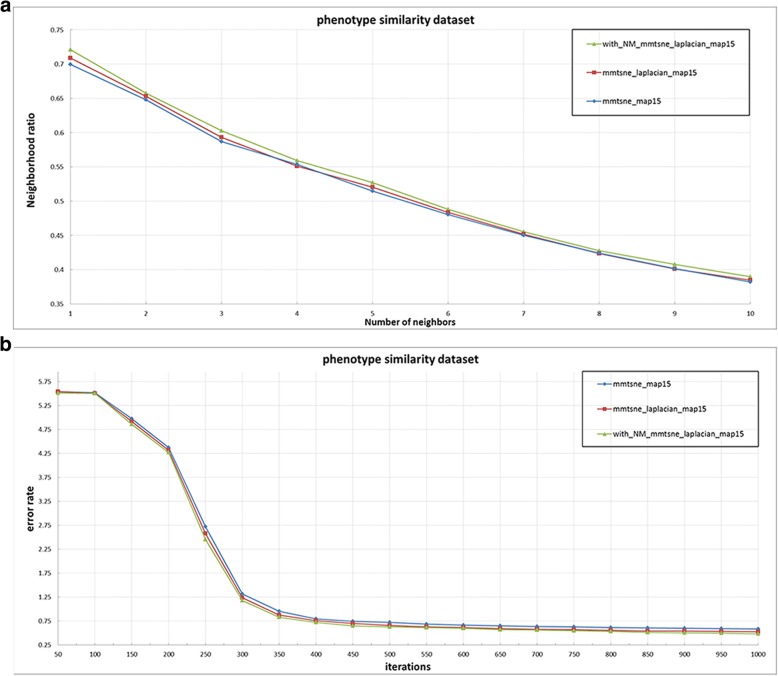
The experimental results of phenotype similarity dataset

**Fig. 3:**
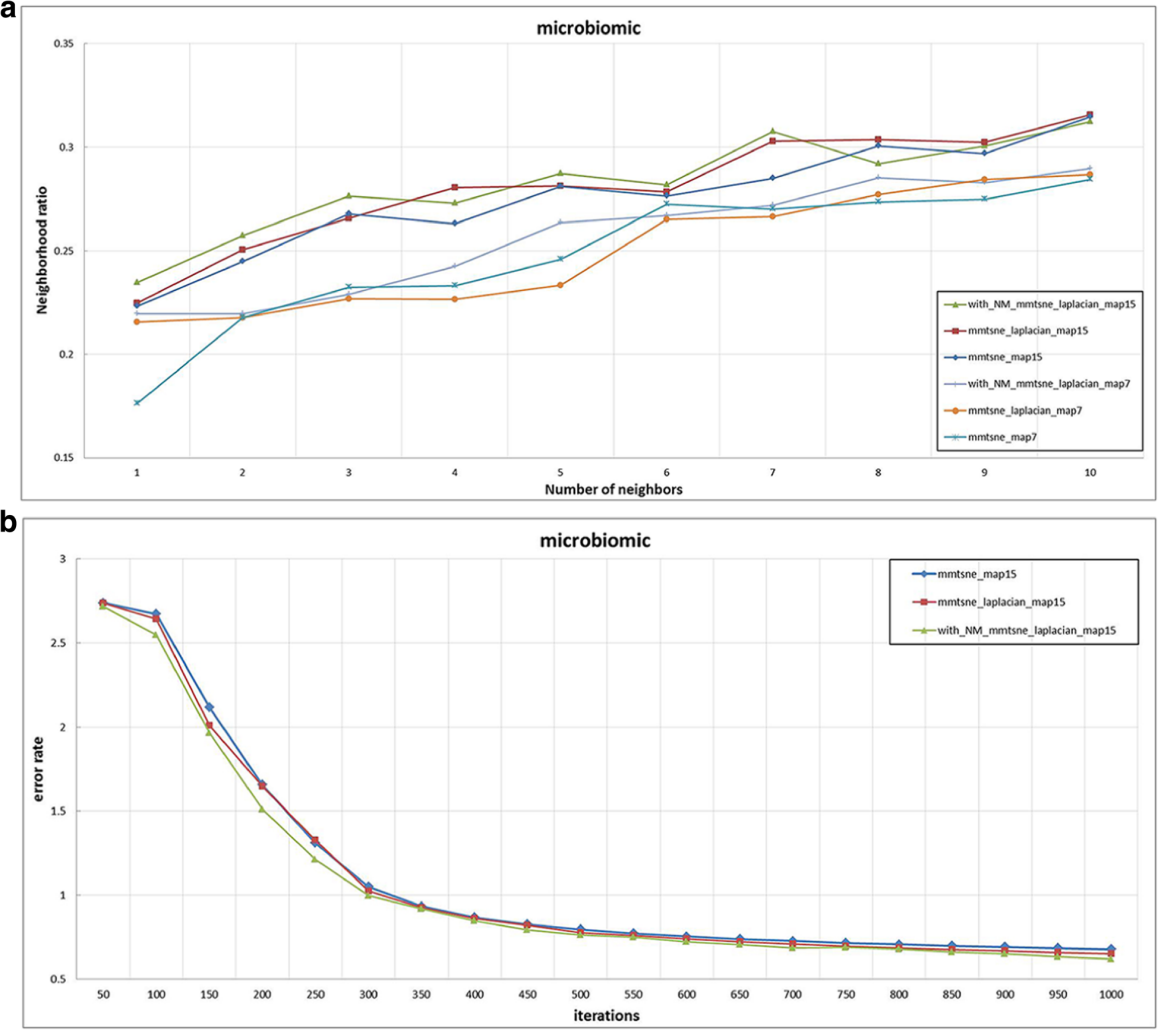
The experimental results of microbiomic dataset

We compare the mm-tSNE regularization based on Nesterov momentum method with the original several mm-tSNE methods in the phenotype (Fig. 2) and microbiome (Fig. 3) dataset respectively using the neighborhood preservation ratio, the error rate and the time complexity as the evaluation indicators.

**Fig. 4:**
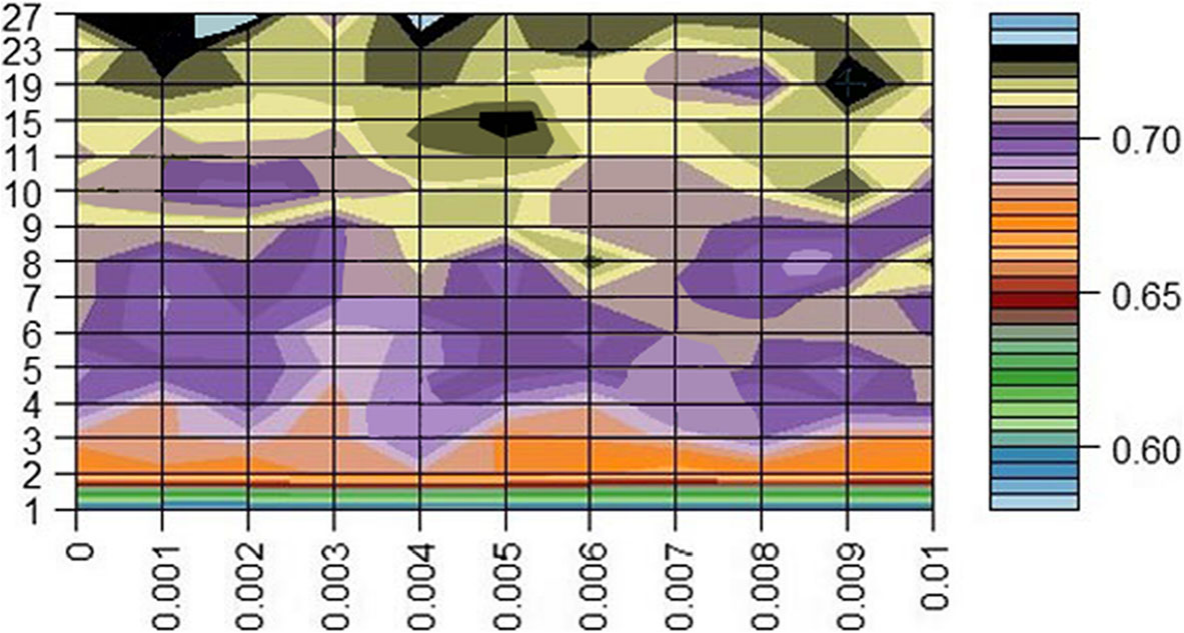
Heatmap of neighbourhood preservation ratio for mm-tSNE regularization based on Nesterov momentum

**Fig. 5:**
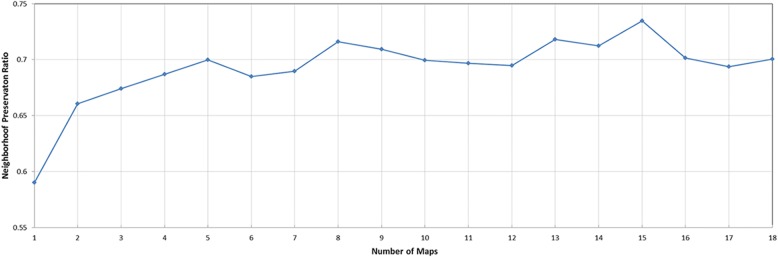
The relationship between NPR and the number of maps. The results show that the relationship between NPR (neighborhood preservation ratio) and increasing number of maps when mm-tSNE regularization based on Nesterov momentum is applied and *λ* = 0.005

**Fig. 6:**
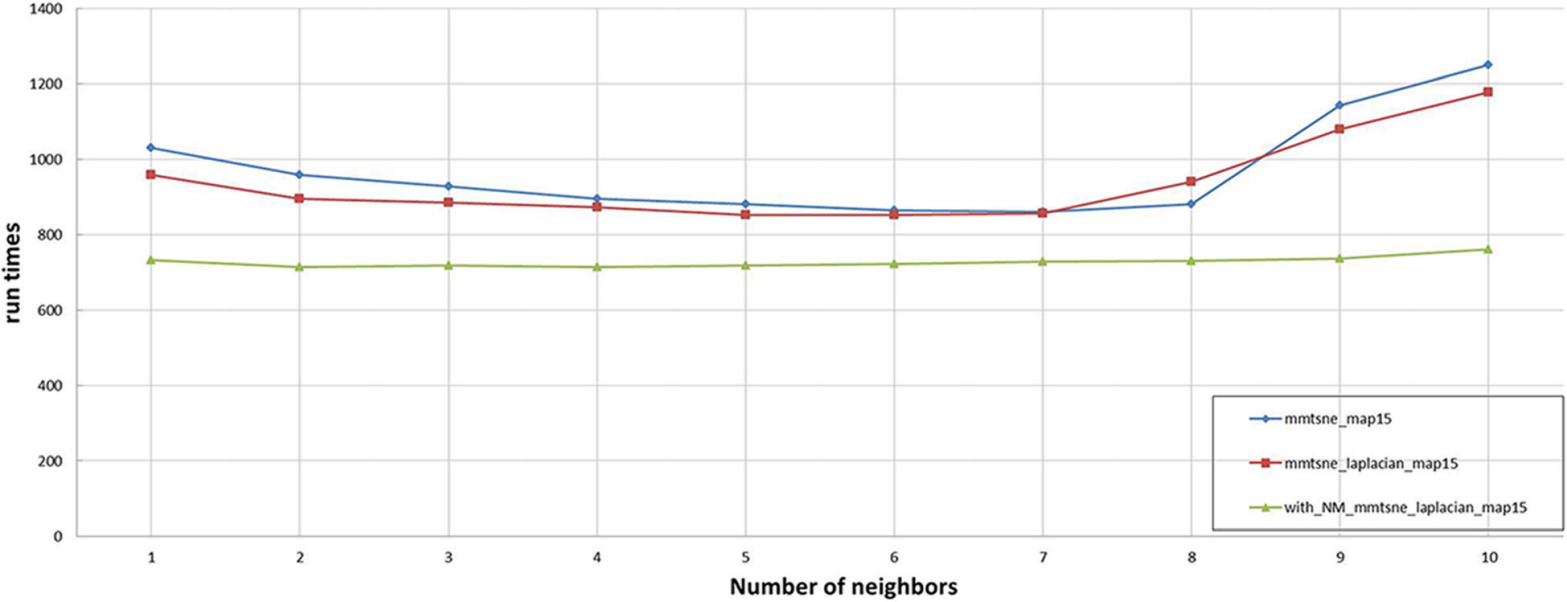
Time complexity comparison results

We then apply the mm-tSNE regularization based on Nesterov momentum to explore the nonmetric relationships on phenotype similarity dataset and microbiomic dataset. The number of model parameters m—the number of maps and λ—the penalty term are selected according to the neighborhood preservation ratio (NPR) (See methods). Fig. 2 and Fig. 3 show the experimental results on phenotype similarity dataset and microbial dataset, respectively. The mm-tSNE regularization based on Nesterov momentum has performance comparable with mm-tSNE and mm-tSNE regularization. The green line in Fig. 2 and Fig. 3 shows that our proposed models are at an advantage over original mm-tSNE methods of several versions. Fig. 4 is the heat map of NPR in the parametric space of m and *λ* when apply mm-tSNE regularization based on Nesterov momentum algorithm. The x-axis represents the value of *λ* in the experiment, and the y-axis represents the number of maps. The color change in the legend represents a gradual decrease in the preservation ratio of the neighborhood from high to low. When *λ* = 0.002 and the number of maps is 27, the neighbor’s preservation ratio is maximized. Nevertheless, according to the experimental results, we choose the number of maps as 15, and set the *λ* as15 as our model parameters, because it is sufficient to model the non-metric structure of phenotype similarities and KOs similarities. When the mm-tSNE regularization based on Nesterov momentum is applied, the relationship between the NPR and the number of maps is shown in Fig.5. When *λ* = 0.005 and *m* = 15, we obtain the highest neighborhood preservation ratio. Overall, the mm-tSNE regularization based on Nesterov momentum obtains better performance compared to other methods and reduces the time complexity of algorithm from Ο(1/*k*) (after *k* steps) to Ο(1/*k*^2^) [21] (See Fig. 6). Since the processed data of the proposed algorithm is a matrix with *N*×*N* size, the spatial complexity of proposed algorithm does not improve relative to the original algorithms. The space complexity of the proposed algorithm is O (*N*^*2*^).

## Discussion

**Fig. 7:**
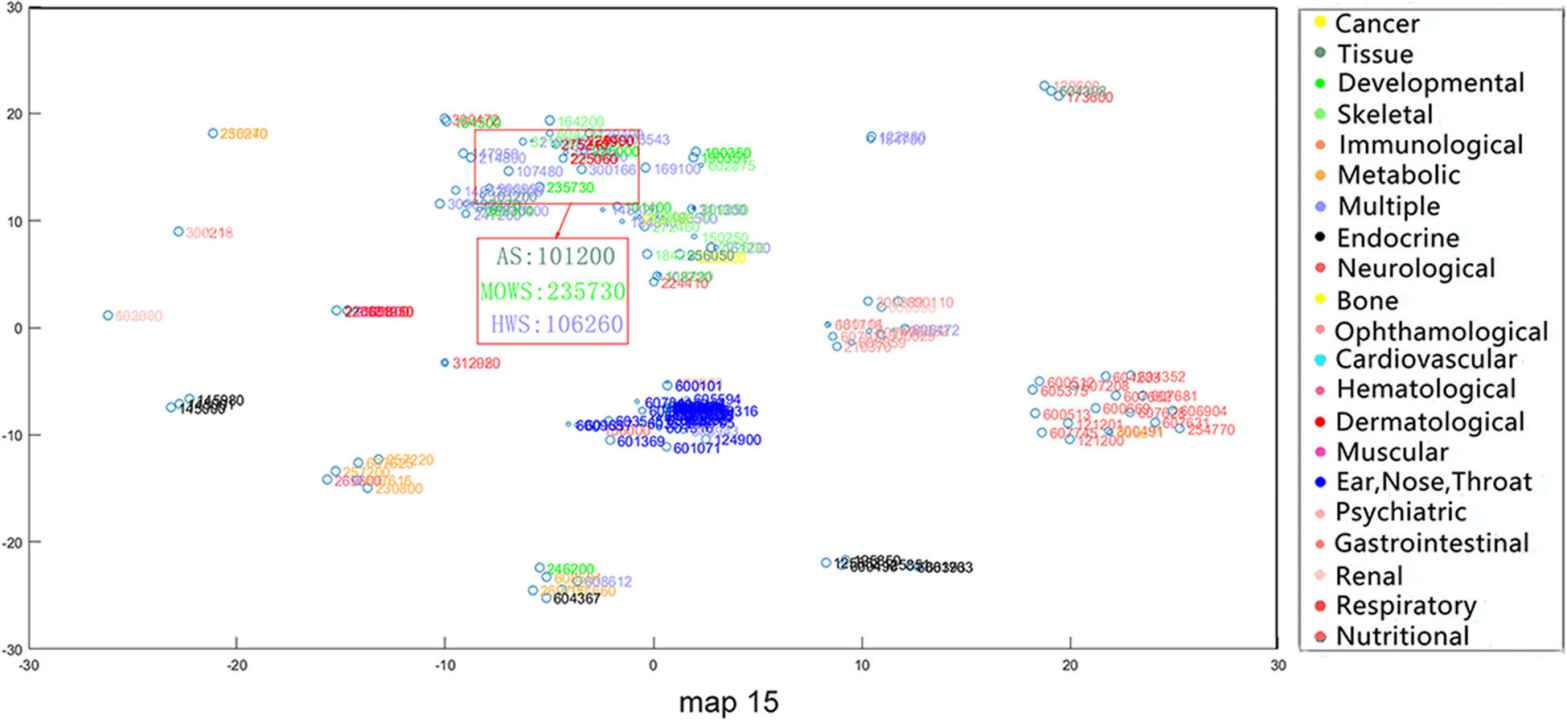
The Map 15 in multiple maps is visualized by the mm-tSNE regularization based on Nesterov momentum method

**Fig. 8:**
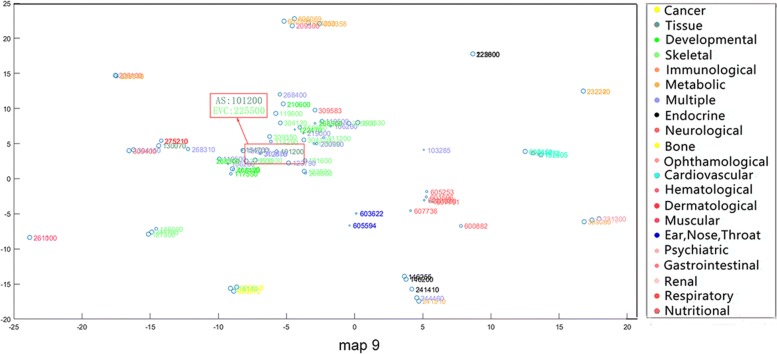
The Map 9 in multiple maps is visualized by the mm-tSNE regularization based on Nesterov momentum method

**Fig. 9:**
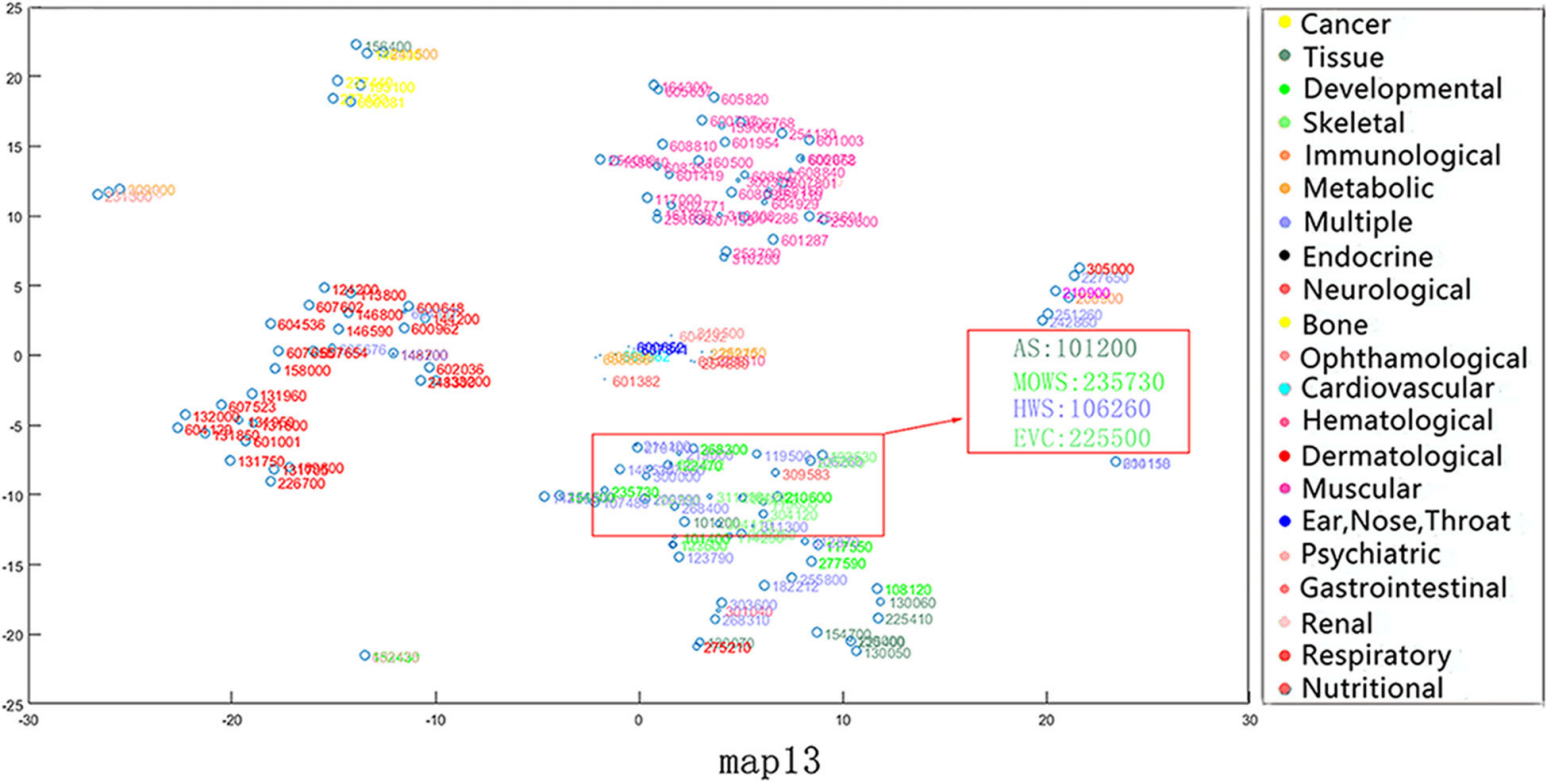
The Map 13 in multiple maps is visualized by the mm-tSNE regularization method

**Fig. 10:**
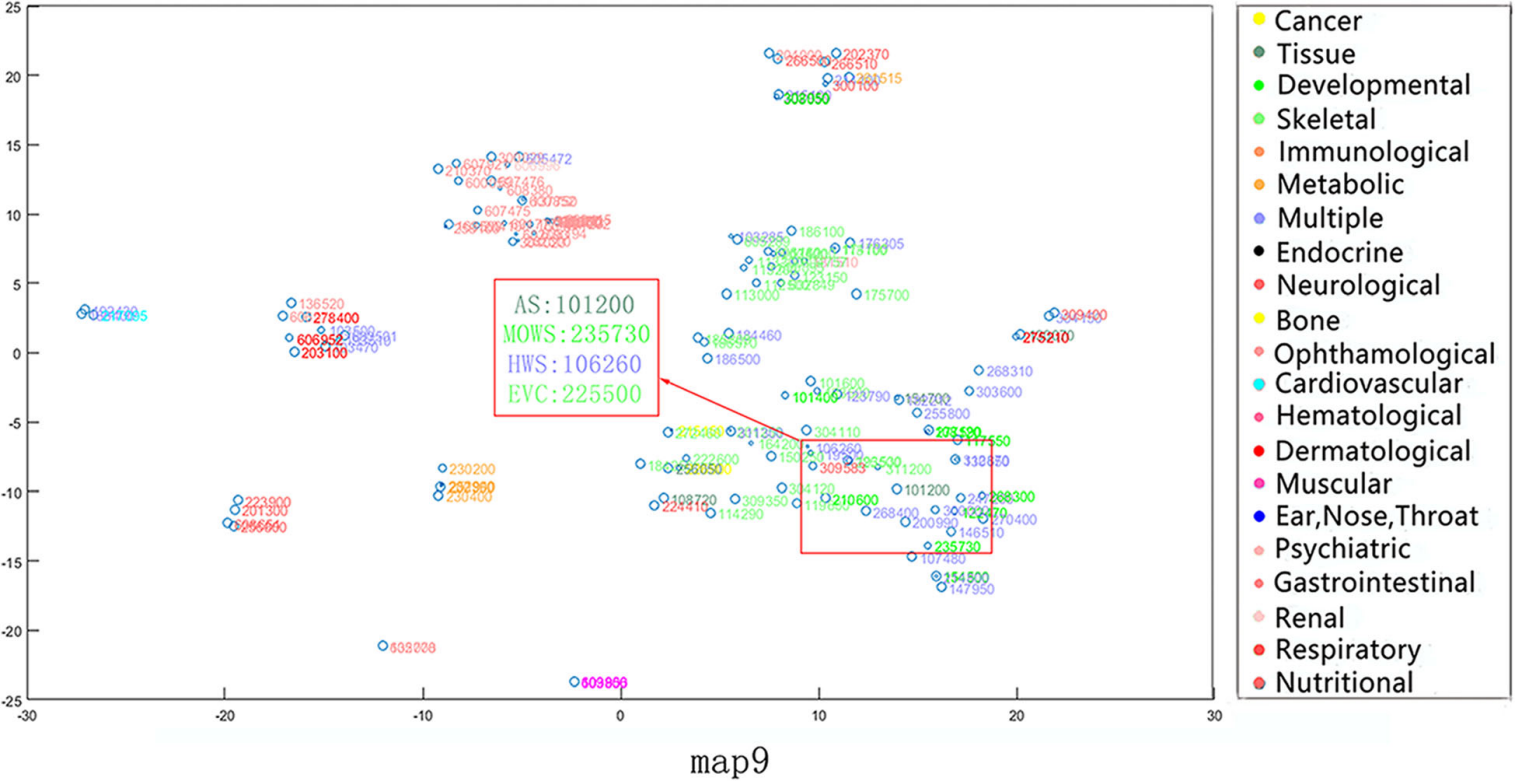
The Map 9 in multiple maps is visualized by the mm-tSNE method

From the phenotypic point of view, similar phenotypes tend to converge into the same class. Nevertheless, some of the phenotypes in the same disease category may exist in other disease categories as well. In addition, we discover that our method compared to mm-tSNE and mm-tSNE regularization can better appropriately model non-transitive similarities between phenotypes. For example, Apert syndrome (AS, OMIM ID: 101200) has importance weights of 0.5967 and 0.3896 at two maps (Maps 9 and 15, See Fig. 7 and Fig. 8). Removing the phenotype of each map with an importance weight less than 0.1 prevents visualization from being too clutter. In Map 9, Ellis-van Creveld syndrome (EVC, OMIM ID: 225500) is one of the neighbors of the AS, with similarity of 0.5148 (See Table 1) and they have an importance weights of 0.5967 and 0.9474 in the metric space Map 9 severally (See Table 2). In Map 15, AS has a near neighbor Mowat-Wilson syndrome (MOWS, OMIM ID: 235730) with similarity 0.5957. From Table 2, it can be found that MOWS is not displayed on Map 9 and EVAS is not displayed on Map 15, the fact that they are both neighbors in single maps. In other words, the neighbor of AS in Map 9 is not essentially the neighbor of it in Map 15. In fact, the similarity between EVC and MOWS is 0 (See Table 1). Although the initial aim of mm-tSNE regularization and mm-tSNE is to find intransitivity similarity. We find that the mm-tSNE and mm-tSNE regularization combine the four phenotypes in Table 1 into one map (See Fig. 9 and Fig. 10). This result indicates that the mm-tSNE regularization based on Nesterov momentum excavates non-transitive similarity of the original several methods without discovering.

**Table 1:** Extracted similarities from original matrix

Phenotype With OMIMID	AS (OMIM:101,200)	MOWS (OMIM:235,730)	HWS (OMIM:106,260)	EVAS (OMIM:225,500)
AS (OMIM:101,200)	1	0.5957	0	0.5148
MOWS (OMIM:235,730)	0.5957	1	0.5298	0
HWS (OMIM:106,260)	0	0.5298	1	0.5392
EVAS (OMIM:225,500)	0.5148	0	0.5392	1

**Table 2:** Importance weights for extracted phenotypes

	Map9	Map15
AS (OMIM:101200)	0.5967	0.3896
MOWS (OMIM:235730)	9.0475e-04	0.9920
HWS (OMIM:106260)	0.1436	0.8348
EVC: (OMIM:225500)	0.9474	0.002

Except MOWS, at Map 15 (see Fig. 7), AS has another near neighbor--Hay-Wells syndrome (HWS, OMIM: 106260) with a similarity 0.5957. AS, MOWS and HWS are all neighbors in Map 15. Nevertheless, astonishing truth is that the similarity between AS and HWS is 0 (See Table 1). Then we have a deep analysis of these three phenotypes. Apert syndrome is a congenital disease; the main symptoms include craniosynostosis, middle facial hypoplasia, hands and feet, with the tendency of bone structure fusion [33–35]. Mowat-Wilson’s syndrome is an autosomal dominant complex dysplasia, characterized by a variety of clinical symptoms such as mental retardation, motor retardation, epilepsy, vasovagal disease and neuropathy, caused by mutations in individual functions [36–38]. HWS is a rare, complex disease characterized by congenital ectodermal dysplasia with a variety of symptoms including thinning hair, mild hypohidrosis, scalp infection, dental hypoplasia, and maxillary dysplasia [39–41]. Although these three diseases belong to different types of diseases (tissue, developmental and multiple respectively), they have the same symptoms, such as nail and tooth dysplasia and skeletal deformities. The experimental result shows that although the text mining method [42] measures the direct similarity between AS and HWS as 0, our method does deduce their true relationship from data. This is different from non-transitive similarity modeling, because they are in the uniform metric space Map 15.

The experimental results demonstrate that our proposed method reveals the non-transitive similarity not found in the original several mm-tSNE methods in microbiomic dataset (See Table 3). K00691 is a maltose phosphorylase involved in glucose metabolism and transcription [43]. Table 3 shows three KOs, of which at least three maps have an importance weight of not less than 0.2, which are respectively close to K00691. K05340 is a transporter involved in signal transduction and glucose uptake of cellular activity. K06204 is a Dnak inhibitor that is involved in the biofilm formation and prokaryotic cell activities of *Escherichia coli* and rRNA transcription [44]. From Table 3 we can see that although these three KOs are similar in Map 7, they are not similar to each other in other maps. For example, K05340 in Map 12 is not similar to K06204. Likewise, K06204 is not similar to K05340 in Map 13. These non-transitive similarities can not be expressed by traditional data visualization methods.

**Table 3:** The weights for KOs similarity. Large values are shown by bold

	Map1	Map2	Map3	Map5	Map7	Map10	Map12	Map13
**K00691**	0.006	0.0041	0.0073	0.0082	**0.2809**	0.0046	**0.2498**	**0.4028**
**K05340**	0.0064	0.0035	0.0056	0.007	**0.3366**	0.0049	**0.5822**	0.0061
**K06204**	0.0030	0.0029	0.0021	0.0934	**0.5417**	0.0031	0.0034	**0.3292**

## Conclusions

We propose a new method to optimize the mm-tSNE regularization cost function. Experimental result shows that this method outperforms several versions of mm-tSNE, when measured by neighborhood preservation rate and error rate. In this study, it is shown that non-metric properties are ubiquitous in biological and microbiological data and should be considered in future studies. Traditional visualization techniques are effective when applied to small and medium-scale data, but they still face a huge challenge when applied to large biological and microbiological data. In future research work, we will propose a method to solve the problem of high computational complexity and problems in data visualization caused by the increase of data volume and the high dimensionality.

## Abbreviations

AS: Apert syndrome
EVC: Ellis-van Creveld syndrome
HWS: Hay-Wells syndrome
mm-tSNE regularization: Multiple maps t-SNE with Laplacian regularization
mm-tSNE: Multiple maps t-SNE
MOWS: Mowat-Wilson syndrome
NPR: Neighborhood preservation ratio
OMIM: Online Mendelian Inheritance in Man
t-SNE: t-Distributed Stochastic Neighborhood Embedding
